# Molecular identification of Giardia lamblia; is there any correlation between diarrhea and genotyping in Iranian population?

**Published:** 2014

**Authors:** Nader Pestechian, Hamidullah Rasekh, Mohammad Rostami-Nejad, Hossein Ali Yousofi, Ahmad Hosseini-Safa

**Affiliations:** 1 Department of Medical Parasitology and Mycology Isfahan University of Medical Sciences, Isfahan, Iran; 2 Central Research Laboratories Isfahan University of Medical Sciences, Isfahan, Iran.; 3 Faculty of Pharmacy, Kabul University of Medical Sciences, Kabul, Afghanistan.; 4 Gastroenterology and Liver Disease Research Center, Shahid Beheshti University of Medical Sciences, Tehran, Iran

**Keywords:** Giardia lamblia, Tpi, PCR-RFLP, Isfahan, Iran

## Abstract

**Aim**: The aim of this study is to investigate the molecular identification of *Giardia lamblia* in patients with diarrhea.

**Background**: Giardiasis caused by *Giardia lamblia* is a common intestinal disease. Although this parasitic infection found in mammals including human, pets and livestock, but few species within the genus *Giardia* can infects humans. *G. lamblia* have seven complex genotypes termed (A-H). Genotype A and B the main causes of human infections.

**Patients and methods**: Sixty seven microscopically positive *G. Lamblia *samples were collected from clinical laboratories in Isfahan province between June 2013 and February 2014. Extraction of genomic DNA was performed for 65 concentrated cysts and 2 cultured trophozoites. Partial sequences of *tpi* including 148-bp and 81-bp were amplified for detection the genotypes A and B using RFLP- PCR protocol respectively.

**Results**: PCR results showed that out of 67 patients with giardiasis infection, genotype A (148 bp) was detected in 40 isolates (59.70%) compared to genotype B (81 bp) isolated was detected in 25 isolates (37.31%). Also two isolates (2.98%) had mix infection infected with genotype A and B. By comparing the frequency of genotype A (81.8%) and genotype B (13.6%), we found that genotype A is six times higher prevalence than genotype B in patients with diarrhea.

**Conclusion**: We suggest that using sensitive techniques and larger sample for detection of *G. lamblia* genotypes and their subtypes would be necessary for investigation the immune system respond and correlation with diarrhea in the future studies in Iran.

## Introduction

*Giardia lamblia*, synonymous with *G. intestinalis* and *G. duodenalis*, is a intestinal flagellant protozoa. Giardiasis caused by *Giardia lamblia* is a common intestinal disease. Usually *G. lamblia* transmitted by contaminated water with cyst (1). Although this parasitic infection found in mammals including human, pets and livestock, but few species within the genus *Giardia* can infect humans (2). Giardiasis is one of the most common intestinal protozoa infections in human that have been reported worldwide (3). In Asia, Africa, and Latin America, about 200 million people have symptomatic giardiasis, with at least 500,000 new cases reported each year (4). *G. lamblia* have seven complex genotypes termed (A-H). Genotypes A and B are the main and only causes of human infections (4, 5). Genotype A have two subtype, subtype AI a main zoonotic subtype and subtype AII belonging to anthroponotic infections, however, in a few studies have been reported in animals. Assemblage B divided to two subtypes named BIII and BIV (1, 2, 6). Genotypic characterization of *G. lamblia* has been shown a useful tool in epidemiological studies or outbreak investigations (7, 8). PCR techniques for genotyping of *G. lamblia* are based on polymorphic genes encoding 18S rRNA, glutamate dehydrogenase (gdh), elongation factor 1-alpha (ef1-α), triose phosphate isomerase (tpi), and β-giardin (5, 9). 

The current study was conducted to determine *G. lamblia* genotypes in Isfahan, using *tpi* markers. PCR-RFLP method was performed based on *tpi*, as this is suitable technique for direct typing of *G. lamblia* in crude specimens. The main purpose of this study was to evaluation the correlation between genotypes of *G. lamblia* and patients with diarrhea.

## Patients and Methods

**Sample collection**

Sixty seventy stool samples from microscopically positive *G. Lamblia c*ysts were collected from clinical laboratories in Isfahan province between June 2013 and February 2014. Samples were collected from both males and females aged 5 months to 70 years. After direct analysis by microscope, the samples without any preservation were kept at 4°C. Cysts of 67 *Giardia*-positive specimens were concentrated from the specimens by flotation on 4 layers (0.5, 0.75, 1, and 1.5 M) and single-layer (0.85 M) sucrose. Among samples, the fresh specimens with a high number of *G. lamblia *(cysts > 205) were isolates and cultured on TYI-S-33 medium (10). Finally, purified cysts and cultured trophozoites were stored at -20°C until further analysis.

**DNA extraction**

Genomic DNA was extracted from 65 concentrated cysts and 2 cultured trophozoites. Those isolates didn’t have suitable DNA were excluded from the study. The trophozoite's DNA was extracted using QIAamp DNA Stool Mini Kit (QIAgen Company, Germany) according to the manufacturer's instruction with some modification and used as template for PCR assay. Extraction of genomic DNA from *Giardia’s *cysts was carried out according to our previous published paper (11).

**PCR amplification**

Partial sequences of *tpi* including 148-bp and 81-bp were amplified for detection the genotypes A and B using RFLP- PCR protocol respectively. For detection of genotype A the following primers were used; forward primer (5´-GGAGACCGACGAGCAAAGC-3´) and reverse primer (5´-CTTGCCAAGCGCCTCAA-3´) and also to identifying genotype B the following primers were used; forward primer (5'-AATAGCAGCACARAACGTGTATCTG-3') and reverse primer (5'-CCCATGTCCAGCAGCATC**-**3') (12, 13).

The reaction mixture for PCR amplification contained 5 μL of 10× buffer (CinnaGen, Iran), 1.5 mM of MgCl2 (CinnaGen, Iran), 0.2 mM of each dNTPs, 1 U of Taq polymerase (CinnaGen, Iran), 20 P mole of each primers and 5–10 μL of extracted template DNA. Amplification of extracted DNA was performed according to the following conditions: One cycle of 94°C for 5 min (initial denaturation), followed by 35 cycles of 20 s at 94°C, 45 s at 60.5°C, 45 s at 72°C, and a final extension at 72°C for 5 min. The DNA sample extracted from cultured trophozoites and *G. Lamblia* standard strain (ATCC: 30888TM) were used as the positive control to monitor PCR success and distilled water was used as the negative control to check for false-positive results that may have arisen from carryover contamination in all experiments. PCR amplicons underwent electrophoresis in a 3% (W/V) agarose gel with Tris-acetate electrophoresis buffer and were stained with ethidium and visualized under a UV trans illuminator. After amplification, the *tpi* gene PCR products for genotype A (148-bp) and B (81-bp), fragment by the enzyme BspLI. 8 μL of PCR product was digested in a total of 20 μL reaction mixture containing 10 U of BspLI (Fermentase EU) and 2 μL of restriction buffer (Tango buffer fermentase). Then the reaction mixture was incubated at 37°C for 2 hours. The digested fragments were fractionated on 3% agarose gel stained by DNA green viewer.

## Results

**Figure 1:**
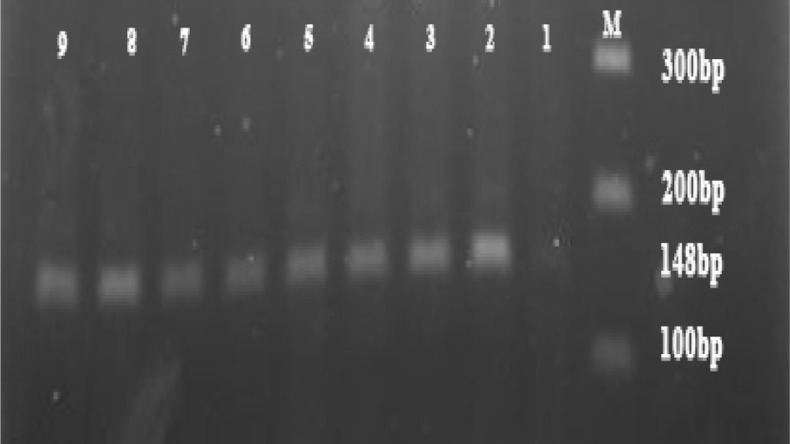
PCR amplification of G. lamblia tpi on 3% agarose gel stained with DNA green viewer. (Lane M: 100 bp gene ruler (fermentase); Line 1: negative control and line 2-9 is belonged to genotype A (148bp).

**Figure 2:**
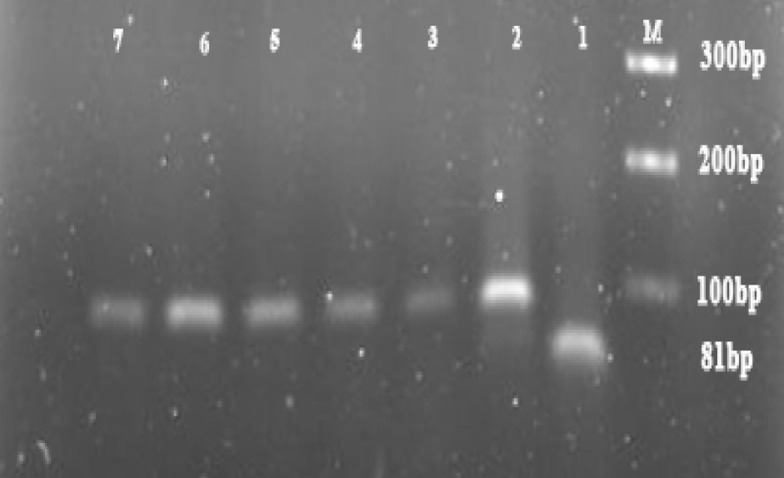
PCR amplification of G. lamblia tpi on 3% agarose gel stained with DNA green viewer. (Lane M: 100 bp gene ruler (fermentase); showed genotype B in line 2 (81bp).

DNA was extracted successfully from all 67 samples and then selected for molecular analysis. PCR results showed that out of 67 patients with giardiasis infection, genotype A (148 bp) was detected in 40 isolates (59.70%) and genotype B (81 bp) was detected in 25 isolates (37.31%) (Figure 1, 2).

Also two isolates (2.98%) had mixed infection with genotype A and B. By comparing the frequency of genotype A (81.8%) and genotype B (13.6%), we found that genotype A is six times higher prevalence than genotype B in patients with diarrhea. However, among patients without diarrhea, frequency of genotype B (82.6%) (17.39%) was significantly higher than genotype A. Statistical Fisher’s test showed, there was significant correlation (P value < 0.05) between patients with diarrhea and genotype A and also asymptomatic infections with genotype B

**Table 1:** Frequency of G. Lamblia genotypes in patients with giardiasis base one clinical sign of diarrhea.

Genotype	Non-diarrheal patients No. (%)	Diarrheal patients No. (%)	Total (%)
genotype A	4(17.39)	36(81.8)	40(59.7)
genotype B	19(82.6)	6(13.6)	25(37.31)
Mix genotype	---	2(4.5)	2(2.98)
Total	23(100)	44(100)	67(100)

## Discussion

Giardiasis in humans is caused by two entirely different genotypes A and B. So far various methods have been performed for molecular diagnosis and detection of *G. lamblia* in the feces and the environment as well. Giardiasis has highly variability symptoms. Some people without any obvious symptoms can be able to dispose infected cysts in feces. It’s not clear why some patients have clinical signs and others are asymptomatic. However, it seems host factors such as immunity conditions and variability in parasite strains, are involved in such differences. Although several studies shown that *G. Lamblia* strains are similar in morphologies but there are different in phenotypic and genetics analysis. In present study we used *tpi* marker and PCR-RFLP method for detection assemblages A and B of *G. lamblia* in stool collection samples from clinical laboratories in Isfahan province. Subtype of *G. lamblia* was not detected in this study. A main purpose of this study was to check the correlation between genotyping of *G. lamblia* and diarrhea in human. The result of this study showed a significant correlation between genotype A of *G. lamblia* and diarrhea. In recent years many studies have been done in the different parts of the World including Bangladesh, Australia, Turkey, Spain, India and Iran and showed the correlation between genotype A and diarrhea (14-20). The same result was reported by Manochehri et al. (2012) in Shahrekord province, Iran (21). In contrast, Rafiei et al. (2013) in Southwest of Iran did not find any correlation between clinical symptoms and assemblages of *G. lamblia *(23). In other study Etemadi et al. (2011) used glutamate dehydrogenase (*gdh*) marker for detect the genotypes of *G. lamblia* by PCR-RFLP method in human faces in Kerman, Southeastern of Iran. They reported both A and B genotype in this region (22). 

On the hand in the study by Hamdan et al. in Saudi Arabia different result to our findings was reported (24). They Used High Resolution Melting (HRM) analysis technique for investigate the correlation between *G. lamblia* genotype and children with and without gastrointestinal symptomatic. They found the correlation between assemblage B and symptomatic patients (24). In support of Hamdan et al. study, Homan et al. in Netherlands and Gelanew et al. in Ethiopia found 100% correlation between the severity of diarrhea and genotype B of *G. lamblia* and obvious correlation between genotype B of *G. lamblia* with sustainable diarrhea respectively (25, 26).The relationship between diarrhea and *G. lamblia* genotypes not essentially means that certain genotypes have higher virulence with diarrhea compared to other genotype. 

 In this study we observe similar diversity in genotypes to other regions in Asia, some parts of Europe and America, though in contrast to some previous studies, we found similar levels of diarrheal symptoms in those individuals infected with assemblage A compared with those infected with assemblage B.

Future research on *Giardia* infection within sensitive technique like HRM analysis technique and using a large sample size could address whether those *Giardia* genotypes found in humans are being transmitted zoonotically and correlation with diarrhea and other clinical sign or not.
